# Kidney Function and Blood and Cerebrospinal Fluid Biomarkers in Alzheimer's Disease and Related Dementias: Systematic Review and Meta-Analysis

**DOI:** 10.1681/ASN.0000001007

**Published:** 2026-01-26

**Authors:** Yuwei Lin, Zaixin Zhao, Nanbo Zhu, Sara Garcia Ptacek, Juan-Jesus Carrero, Annette Bruchfeld, Maria Eriksdotter, Michelle M. Mielke, Nele Brusselaers, Hong Xu

**Affiliations:** 1 Division of Clinical Geriatrics, Department of Neurobiology, Care Sciences and Society, Karolinska Institutet, Stockholm, Sweden; 2 Institute of Advanced Clinical Medicine, Peking University, Beijing, China; 3 Theme Inflammation and Aging, Karolinska University Hospital, Stockholm, Sweden; 4 Department of Medical Epidemiology and Biostatistics, Karolinska Institutet, Stockholm, Sweden; 5 Division of Nephrology, Department of Clinical Sciences, Karolinska Institutet, Danderyd Hospital, Stockholm, Sweden; 6 Department of Nephrology and Health, Medicine and Caring Sciences, Linköping University, Linköping, Sweden; 7 Department of Renal Medicine, Karolinska University Hospital, CLINTEC Karolinska Institutet, Stockholm, Sweden; 8 Department of Epidemiology and Prevention, Wake Forest University School of Medicine, Winston-Salem, North Carolina; 9 Department of Women's and Children's Health, Karolinska Institutet, Stockholm, Sweden; 10 Department of Family Medicine and Population Health, Global Health Institute, University of Antwerp, Antwerp, Belgium; 11 Department of Public Health and Primary Care, Ghent University, Ghent, Belgium

**Keywords:** biomarkers, biostatistics, CKD, chronic kidney disease, dementia, epidemiologic methods, kidney

## Abstract

**Key Points:**

- Lower kidney function is associated with higher concentrations of blood biomarkers for Alzheimer's disease and related dementia.
- Associations between kidney function and cerebrospinal fluid biomarkers for Alzheimer's disease and related dementia were heterogeneous and NS.
- Elevated blood biomarkers for Alzheimer's disease and related dementia in people with impaired kidney function may reflect reduced kidney clearance.

**Background:**

Studies have shown that low kidney function may link to elevated Alzheimer's disease and related dementia fluid biomarkers, but the results are not consistent. This systematic review and meta-analysis aimed to investigate whether kidney function was associated with Alzheimer's disease and related dementia biomarkers (amyloid-*β*, tau, neurofilament light [NfL] protein, and glial fibrillary acidic protein [GFAP]) in blood and cerebrospinal fluid (CSF).

**Methods:**

Human studies were identified through MEDLINE, EMBASE, Cochrane Library, and Web of Science (until May 2, 2025). Studies that reported the association between kidney function and Alzheimer's disease and related dementia biomarkers in blood or CSF among adults were included. Two authors independently screened and extracted data following preferred reporting items for systematic reviews and meta-analyses 2020 guidelines. Descriptive statistics and random-effects meta-analysis were used to analyze pooled effects.

**Results:**

Of the 3024 studies screened, 93 met the inclusion criteria, encompassing 62,503 participants (mean age, 20-96 years; 54% female) from 21 countries. Ninety-one studies reported blood biomarkers, while ten reported CSF biomarkers. In meta-analysis of unadjusted correlation coefficients, kidney function (primarily eGFR) was inversely associated with concentrations of blood NfL, GFAP, Aβ40, β-amyloid 1–40 (A*β*40), β-amyloid 1–42, and phosphorylated tau (p-tau181). In meta-analysis of adjusted regression coefficients, eGFR remained inversely associated with blood biomarker levels. Specifically, every 1 ml/min per 1.73 m^2^ lower eGFR was associated with 0.19 pg/ml higher NfL (95% confidence interval [CI], 0.12 to 0.25), 0.11 pg/ml higher GFAP (95% CI, 0.04 to 0.18), and 0.29 pg/ml higher A*β*40 (95% CI, 0.01 to 0.56). CSF biomarker findings were more heterogeneous and generally null.

**Conclusions:**

Low kidney function was associated with elevated blood biomarkers of Alzheimer's disease and related dementia (NfL, GFAP, and A*β*40), whereas associations with CSF biomarkers were inconsistent and generally null.

## Visual Abstract

**Figure:**
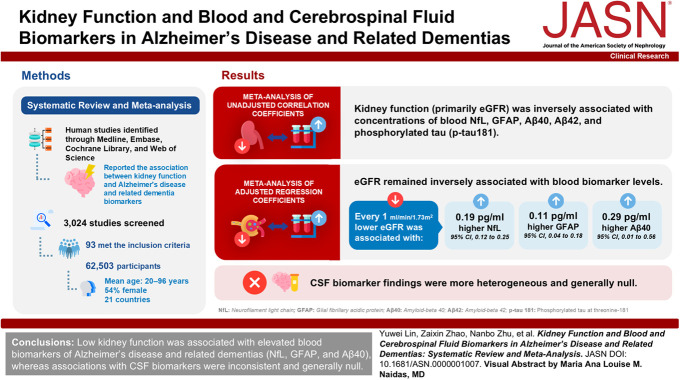

## Introduction

Neurologic disorders are a growing global health concern, particularly in Europe. Dementia, especially Alzheimer's disease, is a major contributor, particularly among older adults.^1^ Dementia leads to progressive cognitive decline and is associated with higher mortality. The number of people with dementia worldwide will rise from 55 million in 2019 to 150 million by 2050.^2^ The National Institute on Aging and Alzheimer's Association has emphasized the inclusion of biomarkers to provide biologic evidence of disease for individuals with cognitive symptoms to aid in the diagnosis of Alzheimer's disease and related dementia.^3^ Biomarkers of Alzheimer's disease pathology include β-amyloid 1–42 (A*β*42), Aβ40, β-amyloid 1–40 (A*β*40), phosphorylated tau (p-tau), and total tau (*t*-tau). Neurofilament light chain (NfL) is a marker of large-caliber axonal degeneration and is a nonspecific biomarker of neurodegeneration. Glial fibrillary acidic protein (GFAP) is a biomarker of brain astrocyte reactivity and neuroinflammation.^4,5^ Initially, these biomarkers were measured in cerebrospinal fluid (CSF). In recent years, technological advances have enabled the increasing use of blood biomarkers, offering a less invasive and more cost-effective alternative.^6^

CKD is common, affecting 10%–15% of the global population, and is characterized by a progressive and irreversible decline in kidney function, typically indicated by a reduced eGFR. CKD has been identified as a risk factor for cognitive decline and dementia; however, it remains unclear whether CKD directly contributes to Alzheimer's disease pathology or whether it increases the risk of dementia through vascular mechanisms or neurodegenerative processes.^7,8^ Although several studies have shown good accuracy of the blood and CSF markers for brain Alzheimer's disease pathology, comorbidities such as CKD can affect the levels of the blood biomarkers due to physiologic reasons.^9^ Studies have suggested that impaired clearance of A*β* in CKD contributes to its accumulation in the brain,^10,11^ while a case report suggested that hemodialysis may reduce brain A*β* burden.^12^ Recent research also suggests that kidney function may be inversely associated with GFAP and NfL concentration in blood.^13,14^ However, findings have been inconsistent, with some studies reporting positive associations or no association.^15,16^

So far, no systematic review and meta-analysis have thoroughly examined the association between kidney function and biomarkers of Alzheimer's disease and related dementia. It remains unclear if CKD contributes to Alzheimer's disease pathology or leads to elevated biomarker levels that result in false positives. In this study, we aim to summarize and evaluate the associations between kidney function indicators and CSF or blood biomarkers using data from existing cross-sectional and prospective human studies. Our study may help clarify whether kidney disease alters biomarker profiles associated with Alzheimer's disease and related dementia and other neurodegenerative disorders.

## Methods

### Search Strategy

A systematic search of human studies published up to May 2, 2025, was conducted in the MEDLINE, Web of Science, Cochrane Library, and EMBASE databases. The search query included the following terms: (*1*) Exposure: eGFR or creatinine or cystatin-C or albuminuria or CKD stages or kidney failure or KRT; (*2*) Outcomes: Alzheimer's disease and related dementia biomarkers (A*β*40, A*β*42, A*β* 42/40 ratio, total tau, p-tau181, p-tau217, p-tau231, GFAP and NfL); (*3*) Biomarker measurement methods: blood or CSF; and (*4*) Population: adults (age ≥18 years). Full search terms for each database are provided in Supplemental Appendix 1. The study protocol has been registered in PROSPERO (ID: CRD420251030761).

### Study Selection, Inclusion, and Exclusion Criteria

Studies included in this systematic review met one of the following criteria: (*1*) measured kidney function as defined by the authors, and reported the association between kidney function and blood or CSF Alzheimer's disease and related dementia biomarkers or (*2*) reported the association between CKD/CKD stages and blood or CSF Alzheimer's disease and related dementia biomarker levels. Importantly, studies were not limited to participants diagnosed with Alzheimer's disease or other neurodegenerative diseases; any study exploring this relationship was considered eligible, regardless of study design. There were no restrictions on language publication. The exclusion criteria were case reports, reviews, book chapters, and study protocols. Studies focused on CSF or blood biomarkers in the context of acute conditions (including AKI, ambulatory patients, or intensive care unit treatment for coronavirus disease 2019) were also excluded. When studies overlapped across databases and reported the same exposure and outcome variables, only the study with the largest sample size was retained.

### Data Extraction

The following data were extracted from the included studies: first author's name, country/region, year of publication, study design, population characteristics, sample size, age, sex, kidney function measurements, biomarkers and specimen (blood or CSF), APOEε4 status, cognitive score, dementia diagnosis, statistical methods, and results related to association between kidney function and Alzheimer's disease and related dementia biomarkers. Covidence systematic review software (Veritas Health Innovation, Melbourne, Australia; www.covidence.org) and EndNote 21 (Clarivate Analytics) were used for deduplication, title and abstract screening, and full-text review. Data screening and extraction were independently conducted by two researchers (Y. Lin and Z. Zhao), with any discrepancies resolved by a third reviewer (H. Xu).

### Exposures and Outcomes

The exposures were kidney function measures, including eGFR, cystatin C, creatinine, and urine albumin-to-creatinine ratio (UACR). eGFR is considered the best overall index of kidney function, typically estimated using serum creatinine and/or cystatin C. Lower eGFR values indicate reduced clearance capacity (higher levels of creatinine or cystatin C) and thus worse kidney function. A higher UACR reflects albuminuria, a marker of kidney damage. CKD definitions varied across studies. Some used full or partial eGFR staging, while others applied a simple CKD/non-CKD classification (using eGFR <60 ml/min per 1.73 m^2^ or International Classification of Diseases codes). Kidney failure was defined as eGFR <15 or the presence of dialysis or kidney transplantation.

The outcomes were blood or CSF Alzheimer's disease and related dementia biomarkers, including A*β*40, A*β*42, A*β* 42/40 ratio, *t*-tau, p-tau181, p-tau217, p-tau231, GFAP, and NfL.

### Statistical Analysis

To examine the relationship between kidney function and Alzheimer's disease and related dementia blood or CSF biomarkers, we extracted all available parameter estimates, including (*1*) correlation coefficients rho (Pearson, Spearman, and partial correlations) from correlation analyses; (*2*) regression coefficients *β* with 95% confidence intervals (CIs), SEs, or *t* values from linear regression models; and (*3*) group means with standard deviations or medians with ranges from *t* tests. For analyses using continuous kidney function measures (eGFR, creatinine, or cystatin-C), no reference group is defined. For categorical exposures (CKD, kidney failure, and eGFR stages), the reference groups were the least severe kidney impairment group (most commonly non-CKD or the highest eGFR category). However, because definitions and analytic approaches differed across studies, incomparable categorical contrasts were summarized narratively. When multiple kidney function assessments were reported, we prioritized eGFR due to its continuous scale and standard adjustment for age and sex. When multiple statistical models with varying adjustments were presented within a study, we prioritized results from the most adequately adjusted model.

We first generated forest plots to visualize correlation coefficients between various kidney function measures and biomarkers. We then created evidence maps summarizing all included studies by statistical significance and direction of association (direct, inverse, or none). The direct association refers to both the kidney function measure and the biomarker changing in the same direction (*e.g*., higher creatinine associated with higher biomarker levels), while the inverse association refers to them changing in opposite directions (*e.g*., lower eGFR associated with higher biomarker levels).

Next, we conducted meta-analyses to quantify associations between kidney function and blood Alzheimer's disease and related dementia biomarkers. Studies were eligible if they reported correlation coefficients or regression coefficients together with 95% CIs, SEs, or *t* values. Studies that reported transformed variables without sufficient information for back conversion or that presented incompatible effect metrics were excluded from the meta-analysis and summarized narratively. To ensure methodologic comparability, Pearson, partial, and Spearman correlation coefficients were not pooled together. Only estimates of the same type were synthesized in each meta-analysis. Specifically, when a study reported effect estimates from independent cohorts or population, each was treated as a separate entry in the meta-analysis. We first pooled correlation coefficients using the Hartung–Knapp–Sidik–Jonkman method with random effects.^17^ We then pooled *β* coefficients from fully adjusted linear regression models. Statistical heterogeneity was quantified using the *I*^2^ statistic from random-effects models.

To explore heterogeneity across observed associations, exposures other than eGFR, such as creatinine and CKD status (yes/no), were included in an exploratory subgroup analysis when at least three comparable studies were available. In addition, we performed an exploratory meta-regression using mean age and female proportion to assess whether these characteristics explained between-study variation. To assess the robustness of our findings, we performed two sensitivity analysis. First, we generated an evidence map by excluding studies published before 2017 to account for improvement in biomarker assay. Second, studies that reported biomarker outcomes on log-transformed scales were included in the meta-analysis; outcomes reported on log_2_ or log_10_ transformations were converted to natural log (ln) to enhance comparability.

Risk of bias due to missing or selectively reported results were assessed only for meta-analyses that includes ≥10 studies. Funnel plots and Egger regression test were used. For meta-analyses with fewer than ten studies, no formal assessments were conducted as standard test for reporting bias are underpowered.^18^

All statistical analyses were performed using the meta ^19^ and forestplot^20^ packages in *R* (version 4.3.1). A *P* value ≤ 0.05 was considered statistically significant.

### Quality Assessment

The 46 studies included in the meta-analysis were evaluated using the Newcastle-Ottawa Scale (NOS) for cross-sectional studies. Quality assessment was conducted independently by two reviewers (Y. Lin and Z. Zhao), with discrepancies resolved through discussion with a third reviewer (H. Xu).

## Results

### Study Selection and Characteristics of Eligible Studies

A total of 3023 records were screened from four databases, and 119 studies were selected for full-text screening. Of these, 26 studies were excluded for the following reasons: case series and reviews,^21–23^ lack of results for the analysis of kidney function and biomarkers,^24–33^ focus on acute conditions,^28,34,35^ or overlapping populations^29,36–42^ (Supplemental Table 1).

**Figure 1:**
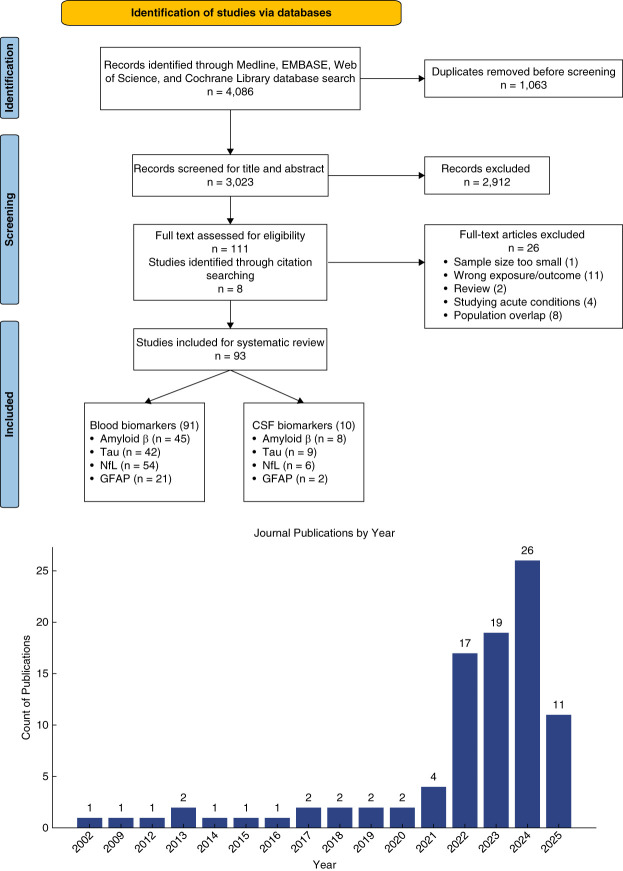
**Preferred Reporting Items for Systematic Reviews and Meta-Analyses (PRISMA) flow chart of study inclusion and year distribution of published studies from 2002 to 2025.** CSF, cerebrospinal fluid; GFAP, glial fibrillary acidic protein; NfL, neurofilament light chain.

Ninety-three studies were included in this systematic review. Of these, 91 involved the association between kidney function and blood biomarkers, while ten involved CSF biomarkers. The studies were published between 2002 and 2025. In total, 62,503 participants from 21 countries were included. Study populations ranged in age from 20 to 96 years, with about 54% female participants. Figure 1 shows the Preferred Reporting Items for Systematic Reviews and Meta-Analyses (PRISMA) flow chart for study selection and distribution of publication years of included studies. Table 1 presents characteristics of studies included in meta-analysis, and details for all 93 studies are presented in Supplemental Table 2.

**Table 1:** Characteristics of studies included in the meta-analysis

Study	Country	Population	*N* (Female %)	Age	Kidney Measures	Biomarkers	Statistical Methods
A.W. Capuano, 2025^43^	US	Participants without dementia	1014 (81%)	79	CKD	p-tau217; NfL; GFAP; A*β*42/A*β*40 (plasma)	Multivariable linear regression
B. Arslan, 2025^44^	Sweden	CU, MCI, AD and non-AD dementia participants	242 (67%)	70 (median)	CKD stages 1–3, eGFR	NfL; GFAP; p-tau181; p-tau217; p-tau231; A*β*40; A*β*42; A*β*42/40 ratio (plasma)	Spearman correlation; multivariable linear regression
D.D. Natale, 2025^45^	Italy	Participants with Fabry disease	40 (58%)	46	Creatinine; cystatin C	NfL (plasma)	Pearson correlation
E. Lee, 2025^46^	South Korea	CU, MCI, AD, and SVCI participants	2935 (30%)	72	eGFR; CKD	p-tau217; NfL; GFAP; A*β*42/A*β*40 (plasma)	Multivariable linear regression
G. Mavraganis, 2025^47^	Greece	Participants with available creatinine levels and Αβ40 measurements	881 (37%)	61	eGFR	A*β*40 (plasma)	Multivariable linear regression
J. Tortosa Carreres, 2025^48^	Spain	Participants with MS	800 (71%; 2933 samples)	Age groups: <40, 40–60, >60	Creatinine; eGFR	NfL (serum)	Spearman correlation
L. Tybirk, 2025^49^	Denmark	Participants with routine tests of creatinine/eGFR and UACR	82	45–50 (range)	eGFR	NfL (plasma)	Spearman correlation
S. De Meyer, 2025^50^	Belgium	AD and non-AD controls matched by age and sex	123 (59%)	68	eGFR	NfL; GFAP (serum)	Spearman correlation
F. Gonzalez Ortiz, 2024^51^	Norway	CU and MCI participants	431 (56%)	64	eGFR	p-tau181, p-tau217, p-tau231 (plasma)	Spearman correlation
F. Motolese, 2024^52^	Italy	Participants with AD	32 (50%)	70	Creatinine	A*β*40, A*β*42, A*β*42/40 ratio, *t*-tau, p-tau181 (plasma, CSF)	Spearman correlation, Mann–Whitney test
J. González Moreno, 2024^53^	Spain	A-ATTR-V30M patients, asymptomatic V30M-TTR mutation carriers, and HC	90 (53%)	51 (median)	eGFR	NfL (serum)	Spearman correlation, multivariable linear regression
J. Sarto, 2024^54^	Spain	Suspected CI and CU participants	360 (55%)	67	eGFR; CKD	p-tau181; GFAP; NfL (plasma)	Multivariable linear regression
J. Wu, 2024^55^	China	Dementia-free participants	1189 (54%)	70 (median)	eGFR	NfL; p-tau181 (plasma)	Multivariable linear regression
K.Y. Kim, 2024^56^	US	MCI and CU participants	720 (49%)	72	eGFR	NfL (plasma)	Multivariable linear regression
L.L. Du, 2024^57^	US	Participants without dementia	424 (68%)	62	eGFR	A*β*42/40 ratio; p-tau181; p-tau217; p-tau231, GFAP, NfL (plasma)	Spearman correlation
T. Axelsson, 2024 (abstract)^14^	Sweden	Participants with CKD stage 3 & 4 and healthy controls	165 (54%)	62	CKD; eGFR	NfL; p-tau231; GFAP (plasma)	Spearman correlation
A. Dittrich, 2023^13^	Sweden	Participants from Gothenburg H70 Birth cohort with and without CKD	744 (51%)	71 (median)	eGFR; CKD	NfL; *t*-tau; p-tau; Aβ42; A*β*40; A*β*42/40 ratio; A*β*42/p-tau (plasma; CSF)	Spearman correlation; Mann–Whitney *U* test
A.H. Simonsen, 2023^58^	Denmark	Participants underwent diagnostic investigations for memory complaints	1190 (47%)	25–96 (range)	eGFR	NfL (serum)	Linear regression
B. Zhang, 2023^59^	US	CU, SMC, MCI, and mild AD participants	645 (46%)	73	eGFR	A*β*42/40; p-tau181; NfL (plasma)	Multivariable linear regression
F. Verde, 2023 (1)^60^	Italy	Participants with ALS and healthy controls	152 (38%)	65	eGFR	GFAP (serum)	Spearman correlation
F. Verde, 2023 (2)^61^	Italy	Participants with AD	30 (60%)	76	eGFR	A*β*42; A*β*40 (plasma)	Spearman correlation
F. Verde, 2023 (3)^62^	Italy	Participants with ALS and healthy controls	255 (42%)	67 (median)	eGFR	NfL (serum)	Spearman correlation
H.L. Sun, 2023^63^	China	Cognitively normal CKD and matched controls with normal kidney function	Plasma sample: 81 (48%); CSF sample: 192 (31%)	Plasma sample: 62; CSF sample: 71	eGFR	A*β*42; A*β*40; *t*-tau (plasma, CSF)	Partial correlation
J.R. Ren, 2023^64^	China	AD and CU controls matched by age and sex	66 (58%)	68	eGFR	A*β*42; A*β*40; *t*-tau; p-tau; NfL (CSF)	Spearman correlation
S.E. O'Bryant, 2023^65^	US	CU participants from HABS-HD	965 (65%)	67	eGFR; CKD	NfL; *t*-tau; A*β*42; A*β*40; A*β*42/40 ratio (plasma)	Partial correlation; ANCOVA
S. Janelidze, 2023^66^	Sweden	Cohort1: participants with MCI; Cohort2: participants with MCI, dementia, and CU controls	Cohort1: 141 (58%); Cohort2: 332 (51%)	Cohort1: 73; Cohort2: 72	eGFR; CKD	p-tau181; p-tau217; tau212-221; tau181-190; pT217/T217; pT181/T181 ratio (plasma)	Multivariable linear regression; Spearman correlation
S. Sedaghat, 2023^10^	US	Participants in ARIC study kidney function measures	2304 (55%)	77	eGFR, ACR	A*β*42; A*β*40; A*β*42/40 ratio (plasma)	Multivariable linear regression
V.K. Ramanan, 2023^67^	USA	Older adults from patient visits in local clinic practice	535 (69%)	80 (median)	CKD	NfL; p-tau181; GFAP; A*β*42/40 ratio (plasma)	Multivariable linear regression
A. Ladang, 2022^68,69^	Belgium	Community-dwelling adults aged over 65 years	409 (58%)	72	eGFR; cystatin C; CKD	NfL (serum)	Partial correlation; Spearman correlation, unadjusted linear regression; Kruskal–Wallis test
E. Rebelos, 2022^70^	Finland	Morbidly obese participants with healthy controls	46 (91%)	45	eGFR	NfL, GFAP (serum)	Multivariable linear regression
J.A. Syrjanen, 2022^71^	US	Participants with MCI, dementia, or CU	996 (44%)	76	CKD	A*β*40, A*β*42, A*β*42/40 ratio, NfL, *t*-tau (plasma)	Multivariable linear regression
J. Traub, 2022 (4)^72^	Germany	Adult patients with chronic stable HF	146 (15%)	64	eGFR, urea	GFAP (serum)	Spearman correlation, linear regression
J. Traub, 2022 (5)^73^	Germany	Adult patients with chronic stable HF	146 (15%)	64	eGFR	NfL; p-tau181 (serum)	Spearman correlation, linear regression
K. Zondra Revendova, 2022^74^	Czech Republic	Participants with MS	60 (75%)	37 (median)	eGFR	NfL (serum)	Linear regression
M.B. Lauvsnes, 2022^75^	Norway	Participants with SLE	67 (87%)	42 (median)	eGFR, creatinine	NfL (plasma)	Multivariable linear regression
R. Gasque Rubio, 2022 (abstract)^76^	Spain	Participants with MS	130	Not reported	Creatinine, eGFR	NfL (serum)	Correlation analysis
R.X. Tang, 2022^77^	US	ADNI: non-AD participants with CKD stage 1–3; VETSA: non-AD participants with CKD stage 1–3, male-male twins	ADNI: 396 (40%); VETSA: 969 (0%)	ADNI: 75; VETSA: 68	Plasma and CSF NfL	Creatinine (plasma)	ADNI: correlation analysis; VETSA: phenotypic correlation
M. Koini, 2021^78^	Austria	Neurologically inconspicuous community-dwelling participants	327 (59%)	65	eGFR	NfL (serum)	Multivariable linear regression
T. Williams, 2021^15^	United Kingdom	Participants with SPMS	122 (67%)	51	eGFR	NfL (serum)	Multivariable linear regression
Y.C. Hou, 2021^16^	Taiwan, China	Hemodialysis patients with/without cognitive impairment	67 (34%)	70	Creatinine; eGFR	NfL (plasma)	Spearman correlation
T. Nakamura, 2018^79^	Japan	Community-based health checkup population	1109 (62%)	54	Creatinine, cystatin C	A*β*40, A*β*42, A*β*42/40 ratio (plasma)	Spearman correlation
W.T. Regenold, 2017^80^	US	Participants meet diagnostic criteria for probable AD, mixed AD, or MCI	21 (24%)	81	Creatinine	A*β*40; A*β*42 (plasma)	Pearson's correlation; ANCOVA
Y.H. Liu, 2015^81^	China	CKD patients with age- and sex-matched normal controls	90 (47%)	62	eGFR, CKD, dialysis	A*β*40, A*β*42 (plasma)	Partial correlation, Kruskal–Wallis tests
A. Ruiz, 2013^82^	Spain	Participants with MCI, AD and CU controls	140 (69%)	71	Creatinine	A*β*40; A*β*42 (plasma)	Pearson's correlation
L. Adbullah, 2009^83^	US	Cognitively normal population	295 (49%)	76	Creatinine	A*β*40 (serum)	Spearman correlation, multivariable linear regression
Z. Arvanitakis, 2002^84^	US	Possible AD and CU controls	279 (60%)	77	Creatinine	A*β*40; A*β*42 (plasma)	Spearman correlations

Country refers to the country where data or cohort are used; sample size refers to population included in analysis exploring kidney and biomarker association; mean age is reported when available; otherwise, median age or age range is presented. Some authors published multiple studies in the same year. To distinguish between them, numbers are listed in parentheses after the author's name and year. These studies are: (*1*) Serum levels of glial fibrillary acidic protein in patients with amyotrophic lateral sclerosis.^60^ (*2*) Influence of kidney function and CSF/serum albumin ratio on plasma Aβ42 and Aβ40 levels measured on a fully automated platform in patients with Alzheimer's disease.^61^ (*3*) Phenotypic correlates of serum neurofilament light chain levels in amyotrophic lateral sclerosis.^62^ (*4*) Serum glial fibrillary acidic protein indicates memory impairment in patients with chronic heart failure.^72^ (*5*) Serum phosphorylated tau protein 181 and neurofilament light chain in cognitively impaired heart failure patients.^73^ Cohorts: ADNI database, Alzheimer's Disease Neuroimaging Initiative database; ARIC, Atherosclerosis Risk in Communities study cohort; HABS-HD, Health and Aging Brain Study-Health Disparities; VETSA, Vietnam Era Twin Study of Aging. Aβ40, β-amyloid 1–40; Aβ42, β-amyloid 1–42; AD, Alzheimer's disease; ALS, amyotrophic lateral sclerosis; ANCOVA, analysis of covariance; ATTR-v, transthyretin variant amyloidosis; CI, cognitively unimpaired; CSF, cerebrospinal fluid; CU, cognitively unimpaired; GFAP, glial fibrillary acidic protein; HC, healthy control; HF, heart failure; MCI, mild cognitive impairment; MS, multiple sclerosis; NfL, neurofilament light chain; SLE, systemic lupus erythematosus; SMC, subjective memory complaint; SPMS, secondary progressive multiple sclerosis; SVCI, subcortical vascular cognitive impairment; UACR, urine albumin-to-creatinine ratio.

### Descriptive Summary and Evidence Mapping of Association

**Figure 2:**
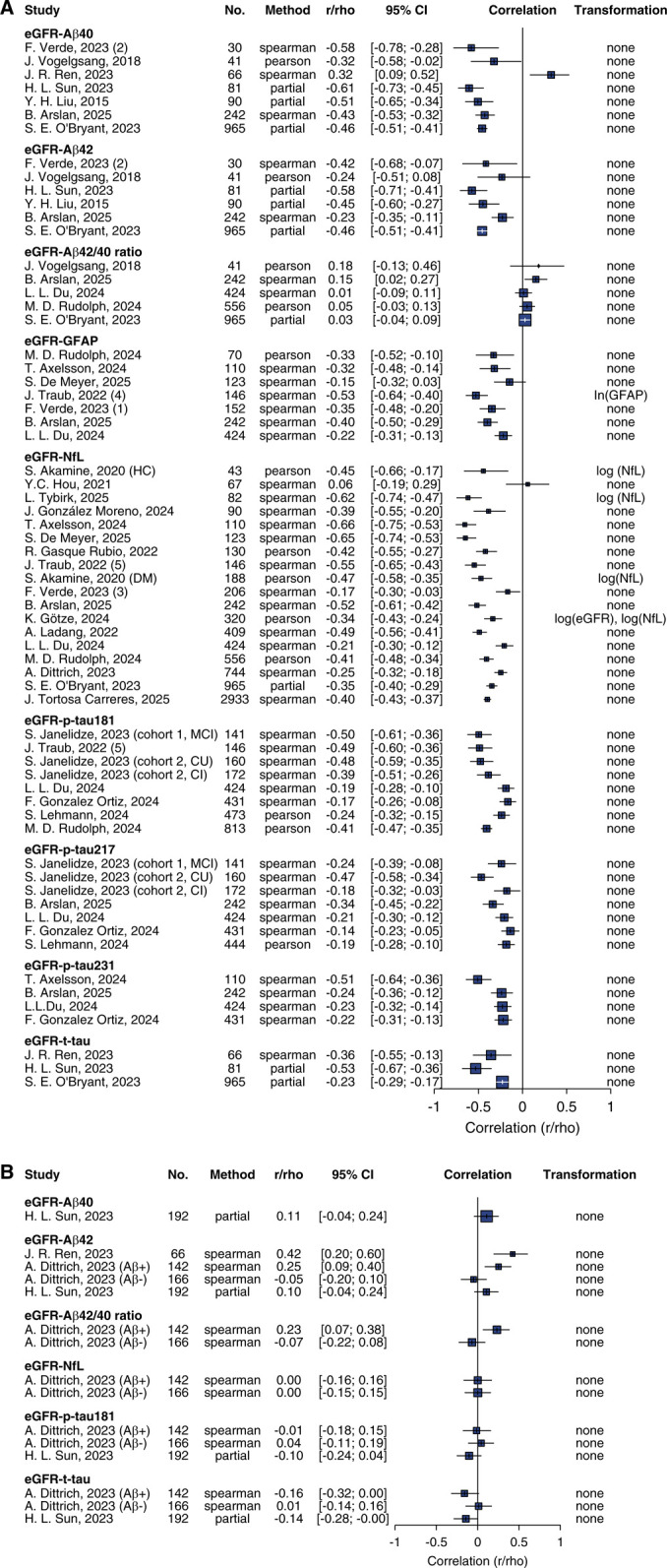
**Nonadjusted correlations between kidney function (eGFR) and Alzheimer's disease biomarkers in blood and CSF.** (A) Blood biomarkers. (B) CSF biomarkers. Study titles: (*1*) Serum levels of GFAP in patients with ALS. (*2*) Influence of kidney function and CSF/serum albumin ratio on plasma A*β*42 and A*β*40 levels measured on a fully automated platform in patients with Alzheimer's disease. (*3*) Phenotypic correlates of serum NfL chain levels in ALS. (*4*) Serum GFAP indicates memory impairment in patients with chronic heart failure. (*5*) Serum phosphorylated tau protein 181 and NfL chain in cognitively impaired heart failure patients. Aβ40, β-amyloid 1–40; Aβ42, β-amyloid 1–42; ALS, amyotrophic lateral sclerosis; CI, confidence interval; p-tau, phosphorylated tau; *t*-tau, total tau.

There were more reported associations between kidney function and blood biomarkers compared with CSF biomarkers, where findings were limited and less consistent (Supplemental Figure 1). eGFR was the most frequently used kidney function measure. Except for A*β*42/40 ratio, eGFR was more commonly reported inversely associated with NfL, GFAP, A*β* proteins, and p-tau in the blood (Figure 2A). By contrast, reported associations between kidney function and CSF biomarkers were less consistent and generally closer to the null (Figure 2B). Detailed *β* coefficients of multivariable models from individual studies are presented in Supplemental Tables 3–9, and corresponding correlation coefficients are shown in Supplemental Figures 2–8.

### Kidney Function and NfL

Studies of blood NfL and kidney function were the most common. Most studies (*n*=29) found an inverse association between eGFR and NfL, while three studies found no association.^15,70,85^ For creatinine, 13 studies showed direct associations with NfL, while three studies showed no association^76,85,86^ and one study reported inverse association.^87^ Five studies reported a direct association between cystatin C and NfL,^45,55,68,88,89^ and in studies evaluating albuminuria, two reported direct associations^14,90^ between UACR and NfL and one reported no association.^89^ All studies comparing individuals with and without CKD found higher blood NfL levels in the CKD group. Findings for CSF NfL were limited and largely null: only one reported a direct association with serum creatinine,^77^ whereas all others reported no association^13,64,68^ (Figure 2B and Supplemental Figure 1B).

**Figure 3:**
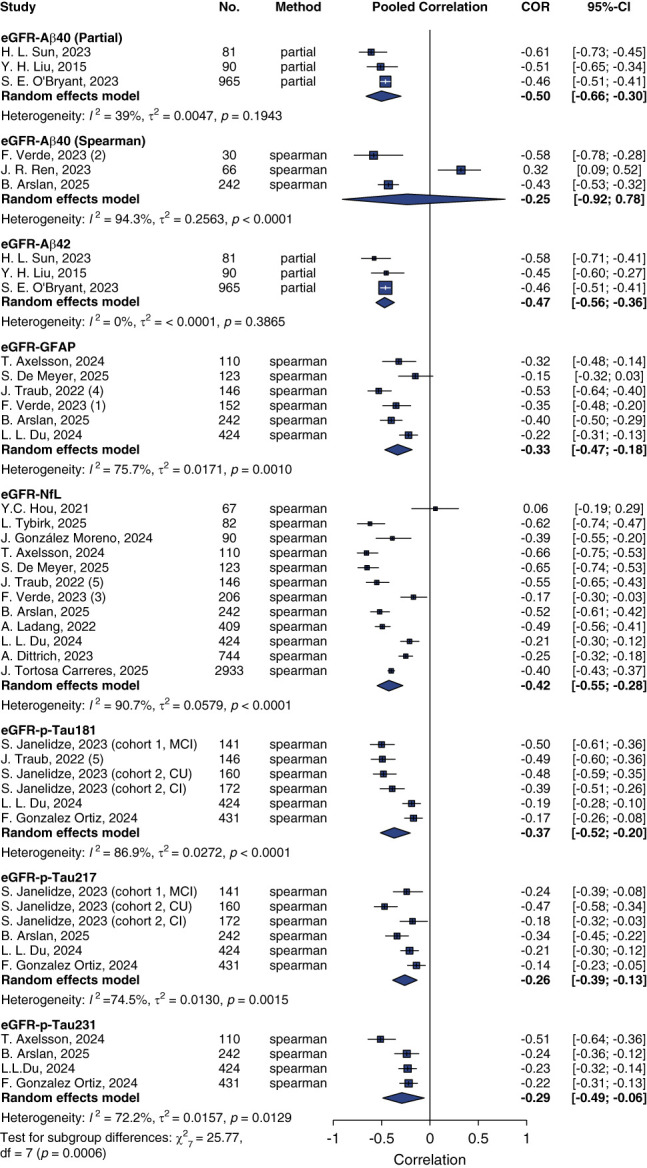
**Forest plot of meta-analysis on association between eGFR and various blood biomarkers using correlation coefficients (rho/r).** Study titles: (*1*) Serum levels of GFAP in patients with ALS. (*2*) Influence of kidney function and CSF/serum albumin ratio on plasma Aβ42 and Aβ40 levels measured on a fully automated platform in patients with Alzheimer's disease. (*3*) Phenotypic correlates of serum NfL chain levels in ALS. (*4*) Serum GFAP indicates memory impairment in patients with chronic heart failure. (*5*) Serum phosphorylated tau protein 181 and NfL chain in cognitively impaired heart failure patients. COR, correlation coefficient.

For quantitative analysis, 12 studies (5576 participants) contributed correlation coefficients to meta-analysis of eGFR and blood NfL (Figure 3).^13,14,16,44,48–50,53,57,62,68^ The pooled correlation was −0.42 (95% CI, −0.55 to −0.28), with substantial heterogeneity (*I*^2^=91%, *P* < 0.001). In exploratory meta-regression of eGFR and blood NfL correlations (k=10), neither mean age (*β*=0.005, *P*=0.79) nor female proportion (*β*=−0.01, *P* = 0.99) explained between-study heterogeneity, and the overall test of moderators was NS (*P* = 0.96). Funnel plot inspection showed no major asymmetry, and the Egger test did not suggest reporting bias (*P*=0.77) (Supplemental Figure 9).

**Figure 4:**
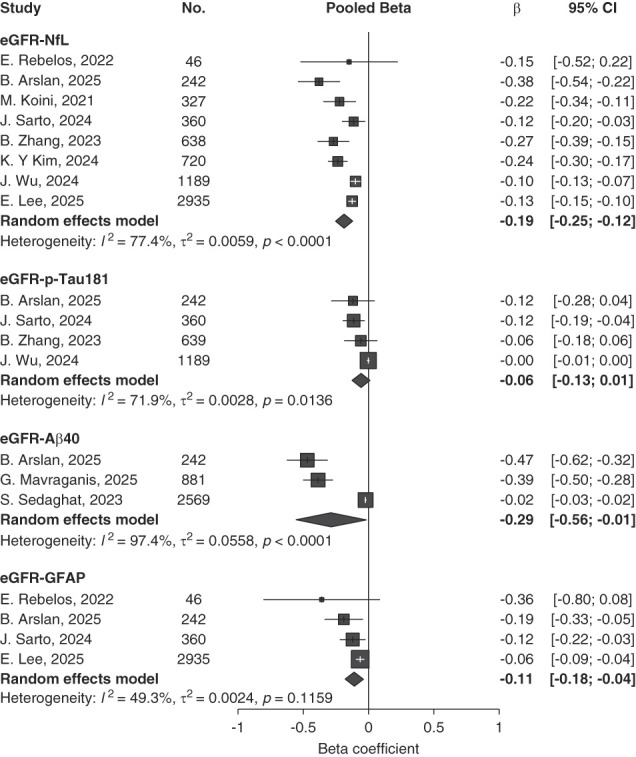
Forest plot of the meta-analysis on the association between eGFR and blood NfL, GFAP, p-tau181, and A*β*40 using beta coefficients.

In meta-analysis pooling *β*-coefficients from eight studies (6457 participants), a similar inverse association was found, and for every 1 ml/min per 1.73 m^2^ lower eGFR, blood NfL concentration was 0.19 pg/ml higher (95% CI, 0.12 to 0.25; Figure 4), with considerable heterogeneity (*I*^2^=75%, *P* < 0.001).^44,46,54–56,70,78^

### Kidney Function and GFAP

Nine studies reported that eGFR was inversely associated with plasma GFAP, while two studies found no association (Figure 2A). We pooled data from five studies (1051 participants) assessing the correlation between eGFR and GFAP using a random-effects model.^14,44,50,57,60^ The results also demonstrated a moderate inverse correlation (rho=−0.29, 95% CI, −0.41 to −0.16; Figure 3). Using adjusted regression coefficients, a meta-analysis of four studies (3583 participants) suggested an inverse association, indicating that for each 1 ml/min per 1.73 m^2^ lower eGFR, GFAP concentration was 0.11 pg/ml higher (95% CI, 0.04 to 0.18^44,46,54,70^; Figure 4). One study examined the associations between CKD stage and GFAP levels in CSF and reported no association (Supplemental Figure 1B).

### Kidney Function and A*β*

Most studies found that worse kidney function was associated with higher blood levels of Aβ42, β-amyloid 1-42 (A*β*42) and A*β*40, showing direct associations with creatinine and CKD stages and inverse associations with eGFR. Findings on the A*β*42/40 ratio were inconsistent. Ten studies reported no association with eGFR,^10,13,44,53,57,59,65,91–93^ while two studies found direct association.^89,94^ All five studies reported no association between CKD status and A*β*42/40 ratio.^5,13,65,67,71^ Mixed results were also found regarding the relationships between creatinine, cystatin C, UACR, advanced CKD stages, kidney failure, and the A*β*42/40 ratio^95^ (Figure 2A, Supplemental Figure 1A, and Supplemental Table 7).

Pooled results from three studies (1136 participants) examined the association between eGFR and blood A*β*42 using partial correlation.^63,65,81^ They showed a strong inverse correlation (rho=−0.47, 95% CI, −0.56 to −0.36), with no significant heterogeneity (*I*^2^=0.0%, *P* = 0.39). Similarly, pooled estimates for the association between eGFR and blood A*β*40, based on both correlation and regression coefficients, indicated a comparable inverse association (rho=−0.50, 95% CI, −0.66 to −0.30; *β*=−0.29, 95% CI, −0.56 to −0.01; Figure 3).

Studies examining the relationship between kidney function and CSF biomarkers were limited. For CSF A*β*42, three studies found no association with eGFR,^63,96,97^ while three reported the direct association.^13,64,98^ CSF A*β*40 also showed mixed results: two studies found no association with creatinine,^52,99^ two found direct associations with eGFR,^64,97^ and one found no association (Figure 2B).^63^

### Kidney Function and Tau

Studies investigating blood *t*-tau mostly reported inverse association with eGFR, and direct association with creatinine and cystatin C. Regarding CKD and kidney failure, studies reported direct correlation with *t*-tau. Kidney function measures showed direct correlations with phosphorylated tau (p-tau) (p-tau181, p-tau217, p-tau231). However, findings for p-tau181 were more mixed, with several studies reporting no association and others showing either direct or inverse relationships (Supplemental Figure 1A).

In the meta-analysis of correlation between eGFR and p-tau181, four studies (1474 participants) showed a moderate inverse association, with rho=−0.37 (95% CI, −0.52 to −0.20) and considerable heterogeneity (*I*^2^=87%, *P* < 0.001; Figure 3).^51,57,66,73^ However, when analyzing the pooled adjusted regression models using *β* coefficients, four studies (2430 participants) found no association (*β* = −0.06, 95% CI, −0.13 to 0.01; Figure 4).^44,54,55,59^ Substantial heterogeneity was also observed (*I*^2^=72%, *P* = 0.01). For p-tau217 and p-tau231, only meta-analyses of correlation coefficients were conducted. Both showed significant inverse associations with eGFR, with pooled correlation coefficients of −0.26 (95% CI, −0.39 to −0.13) for p-tau217^44,51,57,66^ and −0.29 (95% CI, −0.49 to −0.06) for p-tau231^14,44,51,57^ (Figure 3).

Fewer studies examined the relationship between kidney function and CSF tau. For *t*-tau, four studies found an inverse correlation with eGFR, while one reported no association. Five studies also reported no association between creatinine or CKD and *t*-tau. For p-tau, four studies found no association between eGFR and p-tau181, while two studies reported the inverse association. Studies on creatinine and CKD also reported no association with p-tau181 (Figure 2B, Supplemental Figure 1B, and Supplemental Table 9).

### Sensitivity Analysis

Eight studies published before 2017 were excluded from the sensitivity analysis. The excluded studies predominantly reported associations between creatinine and A*β* biomarkers and were conducted before the adoption of improved blood biomarker assays. The remaining studies were presented in an evidence map, with a figure consistent with that of the main analysis (Supplemental Figures 1A and 10).

For creatinine and blood A*β* biomarkers, pooled Spearman correlation showed a positive association with A*β*40 (rho=0.26, 95% CI, 0.12 to 0.39)^52,79,83,84^ but not with A*β*42 (rho=0.14, 95% CI, −0.27 to 0.50).^52,79,84^ The pooled Pearson correlation for A*β*40 was NS, and no significant association was observed between creatinine and NfL (*r*=0.29, 95% CI, −0.15 to 0.63^45,76,77^; Supplemental Figure 11). In meta-analysis of A*β*42/40 ratio (untransformed outcome), no significant association with CKD was observed (*β*=0.08; 95% CI, −0.00 to 0.17).^43,67,71^ In meta-analysis with log-transformed outcomes, CKD was positively associated with GFAP (*β*=0.20; 95% CI, 0.08 to 0.32)^43,67^ and NfL (*β*=0.52; 95% CI, 0.33 to 0.71)^43,67^ (Supplemental Figures 12 and 13).

### Quality Assessment

The quality of the studies included in the meta-analysis was assessed through NOS, adapted for cross-sectional studies. A total of 46 studies were included, with scores ranging from 5 to 8. Specifically, 20 studies were rated as “Satisfactory” and 26 as “Good,” and none were rated as “Unsatisfactory” by either reviewer (Supplemental Table 10).

## Discussion

This systematic review and meta-analysis synthesized the current evidence on the association between kidney function and blood and CSF biomarkers of Alzheimer's disease and related dementia. Across 93 studies and over 62,000 participants, we found generally consistent evidence suggesting a potential correlation between lower kidney function and elevated concentrations of biomarkers in blood, especially NfL and GFAP. By contrast, findings from CSF biomarkers (10 studies and 3951 participants) were more heterogeneous, with greater proportion of associations around the null.

Our results showed that eGFR was inversely associated with blood NfL in both the crude analysis (pooled correlation coefficients) and the adjusted analysis (pooled *β* coefficients). Similar associations were observed when using creatinine and CKD,^55,75,77,89,96,99–103^ indicating that worse kidney function was associated with higher blood NfL concentration. This implies that impaired kidney function may be associated with blood biomarkers of neurodegeneration. However, the possibility that reduced kidney clearance contributes to higher circulating NfL concentrations cannot be excluded. Because of limited evidence, it remains unclear whether elevated blood NfL levels in patients with kidney insufficiency reflect neuronal damage.

GFAP is an intermediate filament in the astrocyte cytoskeleton and can be used as a marker of neuroinflammation. Our meta-analysis showed an inverse association between eGFR and blood GFAP level. Some studies have shown a sex difference in this association, with higher GFAP levels reported in women with CKD.^104^ Unfortunately, the number of studies reporting data on sex differences was too few to conduct a sex-specific analysis in our study. Future studies should investigate the mechanisms underlying kidney dysfunction and neuroinflammation and explore sex-specific differences.

Although the findings on kidney function and A*β* were mixed, worse kidney function was generally associated with higher blood A*β*42 and A*β*40 levels. By contrast, the blood A*β*42/A*β*40 ratio showed more consistent findings, with most studies indicating no significant link to kidney function.^57,59,61,65,91,93,94,97^ Given that the CSF A*β*42/A*β*40 ratio is a well-established Alzheimer's disease biomarker, our results indicate that the blood A*β*42/A*β*40 ratio also appears stable and is not significantly influenced by kidney function. The observed stability of blood A*β*42/A*β*40 ratio supports its potential as a noninvasive biomarker of Alzheimer's disease pathology.

For tau biomarkers (*t*-tau and p-tau), most studies found that reduced kidney function was associated with elevated levels of blood tau biomarkers. However, our meta-analysis focused specifically on p-tau181 due to data limitation. While the pooled crude correlation indicated a significant association between lower eGFR and higher blood p-tau181, the pooled adjusted *β* coefficient showed that this association was not statistically significant. Several studies have reported an association between CKD (defined as eGFR <60 ml/min per 1.73 m^2^) and blood p-tau181.^5,41,66,67,105^ Our meta-analysis instead used eGFR as a continuous variable, which may dilute the effect of CKD on p-tau181, as small changes in eGFR do not always relate to noticeable differences in p-tau181 concentrations.

Our finding suggests the need to incorporate kidney function into biomarker interpretation. A better understanding of how reduced kidney function relates to biomarker concentrations may clarify whether diagnostic thresholds need to be adapted for people with impaired kidney function. While this question remains unclear, incorporating kidney function indicators into biomarker-based diagnostic algorithms may become an important step toward more personalized and accurate clinical assessment.

This is the first systematic review and meta-analysis examining the relationship between kidney function and Alzheimer's disease and related dementia biomarkers. We included more than 62,000 participants, integrated various kidney function markers, analyzed studies from different populations and disease groups, and evaluated a wide range of biomarkers.

This study has some limitations. First, although we included a large number of studies, substantial methodologic heterogeneity was observed, including differences in biomarker assays, data transformations, and statistical models, which limited the number of studies that could be pooled in meta-analysis. Second, there was considerable variation in how kidney function was categorized across studies, such as CKD versus non-CKD, different eGFR cutoffs, and kidney failure definitions), which further limited quantitative synthesis of categorical comparisons. Although we attempted to contact authors for detailed reports, studies from which we could not obtain such information were excluded. In addition, we lacked data on specific factors such as sex, age, and APOEε4 status, which may modify the relationship between kidney function and Alzheimer's disease and related dementia biomarkers. Finally, although several of the included studies were cohort-based, most reported only cross-sectional associations between kidney function and Alzheimer's disease and related dementia biomarkers. As a result, the temporal ordering between exposure and outcome could not be established, and the extent to which declining kidney function may causally influence biomarker levels remains uncertain. This limitation reduces our ability to infer causal relationships and highlights the need for longitudinal studies with repeated biomarker assessments.

Our systematic review and meta-analysis comprehensively evaluated the association between kidney function and Alzheimer's disease and related dementia biomarkers in blood and CSF. Low kidney function was associated with higher concentrations of Alzheimer's disease and related dementia biomarkers, especially NfL and GFAP. The relationship between CSF biomarkers and kidney function showed inconsistency, with varying results across studies. These findings suggest that lower kidney function may be linked to Alzheimer's disease and other neurodegenerative conditions. However, the observed higher level of Alzheimer's disease and related dementia biomarkers in individuals with impaired kidney function should be interpreted with caution, as it may be influenced by reduced kidney clearance rather than reflecting a true neurodegenerative process. Our finding highlights the need for further research to clarify the underlying mechanisms linking kidney function and these biomarkers. Future studies should use standardized methodologies, longitudinal designs, and larger cohorts to improve the robustness of evidence. Understanding these associations is significant for refining biomarker interpretation in individuals with kidney dysfunction and developing personalized diagnostic strategies for Alzheimer's disease and related dementia.

## Data Availability

The study is based on data extracted from previously published studies. A complete list of studies with DOIs is provided in the reference list. No individual-level or unpublished data were used.

## Supplemental Material

Supplemental Table 1. Excluded studies in full-text screening.

Supplemental Table 2. Characteristics of included studies.

Supplemental Table 3. The association between kidney function and blood NfL level expressed by beta coefficients.

Supplemental Table 4. The association between kidney function and blood GFAP level expressed by beta coefficients.

Supplemental Table 5. The association between kidney function and blood amyloid *β*40 level expressed by beta coefficients.

Supplemental Table 6. The association between kidney function and blood amyloid *β*42 level expressed by beta coefficients.

Supplemental Table 7. The association between kidney function and blood amyloid *β*42/40 ratio expressed by beta coefficients.

Supplemental Table 8. The association between kidney function and blood tau (*t*-tau, p-tau181, p-tau217, p-tau231) levels expressed by beta coefficients.

Supplemental Table 9. The association between kidney function and CSF Alzheimer's disease and related dementia biomarkers levels expressed by beta coefficients.

Supplemental Table 10. Quality assessment based on NOS.

Supplemental Figure 1. Evidence mapping of association between kidney function and Alzheimer's disease and related dementia biomarkers in blood and CSF.

Supplemental Figure 2. Forest plot showing the correlation coefficients for the association between kidney function and blood NfL level.

Supplemental Figure 3. Forest plot showing the correlation coefficients for the association between kidney function and blood GFAP level.

Supplemental Figure 4. Forest plot showing the correlation coefficients for the association between kidney function and blood amyloid *β*40 level.

Supplemental Figure 5. Forest plot showing the correlation coefficients for the association between kidney function and blood amyloid *β*42 level.

Supplemental Figure 6. Forest plot showing the correlation coefficients for the association between kidney function and blood amyloid *β*42/40 ratio.

Supplemental Figure 7. Forest plot showing the correlation coefficients for the association between kidney function and blood tau level (p-tau181, p-tau217, p-tau231, *t*-tau).

Supplemental Figure 8. Forest plot showing the correlation coefficients for the association between kidney function and CSF Alzheimer's disease and related dementia biomarkers.

Supplemental Figure 9. Funnel plot of eGFR-NfL correlation meta-analysis.

Supplemental Figure 10. Evidence mapping of association between kidney function and Alzheimer's disease and related dementia biomarkers in blood, excluding studies published before 2017.

Supplemental Figure 11. Meta-analysis of association between serum creatinine and blood A*β*42, A*β*40, and NfL with correlation coefficients.

Supplemental Figure 12. Meta-analysis of association between CKD (yes/no) and blood A*β*42/40 ratio with *β* coefficients.

Supplemental Figure 13. Meta-analysis of association between kidney function and log transformed blood biomarkers with *β* coefficients.

Supplemental Appendix 1. Search strategy for MEDLINE, EMBASE, Web of Science, and Cochrane Library.
